# The structure and functional profile of ruminal microbiota in young and adult reindeers (*Rangifer tarandus*) consuming natural winter-spring and summer-autumn seasonal diets

**DOI:** 10.7717/peerj.12389

**Published:** 2021-11-24

**Authors:** Elena Yildirim, Larisa Ilina, Georgy Laptev, Valentina Filippova, Evgeni Brazhnik, Timur Dunyashev, Andrey Dubrovin, Natalia Novikova, Daria Tiurina, Nikolay Tarlavin, Kasim Laishev

**Affiliations:** 1 Molecular Genetic laboratory, BIOTROF+ LTD, Saint-Petersburg, Russia; 2 Department of Animal Husbandry and Environmental Management of the Arctic, Federal Research Center of Russian Academy Sciences, Pushkin, Saint-Petersurg, Russia

**Keywords:** Reindeer, Rumen microbiome, Arctic zone, NGS, PICRUSt2

## Abstract

**Background:**

The key natural area of Russian reindeer (*Rangifer tarandus*, Nenets breed) is arctic zones, with severe climatic conditions and scarce feed resources, especially in the cold winter season. The adaptation of reindeer to these conditions is associated not only with the genetic potential of the animal itself. The rumen microbiome provides significant assistance in adapting animals to difficult conditions by participating in the fiber digestion. The aim of our study is to investigate the taxonomy and predicted metabolic pathways of the ruminal microbiota (RM) during the winter–spring (WS) and summer–autumn (SA) seasons, in calves and adult reindeer inhabiting the natural pastures of the Yamalo-Nenetsky Autonomous District of the Russian Federation.

**Methods:**

The RM in reindeer was studied using the Next Generation Sequencing method with the MiSeq (Illumina, San Diego, CA, USA) platform. Reconstruction and prediction of functional profiles of the metagenome, gene families, and enzymes were performed using the software package PICRUSt2 (v.2.3.0).

**Results:**

The nutritional value of WS and SA diets significantly differed. Crude fiber content in the WS diet was higher by 22.4% (*p* < 0.05), compared to SA, indicating possibly poorer digestibility and necessity of the adaptation of the RM to this seasonal change. A total of 22 bacterial superphyla and phyla were found in the rumen, superphylum Bacteroidota and phylum Firmicutes being the dominating taxa (up to 48.1% ± 4.30% and 46.1% ± 4.80%, respectively); while only two archaeal phyla presented as minor communities (no more then 0.54% ± 0.14% totally). The percentages of the dominating taxa were not affected by age or season. However, significant changes in certain minor communities were found, with seasonal changes being more significant than age-related ones. The percentage of phylum Actinobacteriota significantly increased (19.3-fold) in SA, compared to WS (*p* = 0.02) in adults, and the percentage of phylum Cyanobacteria increased up to seven-fold (*p* = 0.002) in adults and calves. Seasonal changes in RM can improve the ability of reindeer to withstand the seasons characterized by a low availability of nutrients. The PICRUSt2 results revealed 257 predicted metabolic pathways in RM: 41 pathways were significantly (*p* < 0.05) influenced by season and/or age, including the processes of synthesis of vitamins, volatile fatty acids, and pigments; metabolism of protein, lipids, and energy; pathogenesis, methanogenesis, butanediol to pyruvate biosynthesis, cell wall biosynthesis, degradation of neurotransmitters, lactic acid fermentation, and biosynthesis of nucleic acids. A large part of these changeable pathways (13 of 41) was related to the synthesis of vitamin K homologues.

**Conclusion:**

The results obtained improve our knowledge on the structure and possible metabolic pathways of the RM in reindeer, in relation to seasonal changes.

## Introduction

Cervidae is the second largest family in suborder Ruminantia of order Artiodactyla, inhabiting different geographic and climatic zones of the Earth, from the Arctic tundra to the tropic forests (Hassanin et al., 2012). The reindeer (*Rangifer tarandus* L.) is the single species of this family with circumpolar area involving boreal, tundral, sub-arctic, arctic, and alpine zones of the northern parts of Asia, Northern America, and Europe. It is, interestingly, also the single entirely domesticated species of the Cervidae, serving as a source of meat, milk, and fat, skins, and other nutritionally and/or therapeutically valuable products. The meat and milk of reindeer have dietetic properties, as the meat is low-fat and contains high amounts of protein and vitamins, while the milk contains more protein and less lactose, compared to cow milk (Young & Park, 2006). Reindeer have served as the basis of the economic set-up of Arctic peoples for centuries, helping in their adaptation to the severe climatic conditions.

The Arctic zone of the Russian Federation, one of the key natural areas of the reindeer, is one of the severest places in the world, with the lowest temperatures and levels of available plant biomass. Over half of the Yamalo-Nenetsky Autonomous District (YNAD, where our study was performed) lies within the Arctic Cycle. The climate is determined by the presence of permafrost, the vicinity of the cold Kara Sea, long winters (up to 8 months), short summers, and strong winds. Low ambient temperatures pose an environmental challenge affecting the physiology and metabolism of reindeer. In addition, the formation of a snow crust in winter frequently results in periods of acute starvation for the reindeer, limiting the growth of their populations; for example, the formation of vasts snow crust areas in the YNAD in the winter of 2008 resulted in the death of ca. 50,000 reindeer, while that in the winter of 2016 resulted in the death of ca. 100,000 animals (Zabrodin et al., 2019).

Practical reindeer farming in Northern regions is usually based on natural pastures without supplementary feeding; hence, the reindeer are strongly dependent on the seasonal availability of feedstuffs (Pettorelli et al., 2005). Seasonal changes in the availability and quality of feedstuffs for reindeer are usually drastic. The basis of their summer diets are higher plants (Embryophyta) while, in winter, the reindeer consume mixed diets with a prevalence of lichens. Due to alterations in the chemical composition of these feed sources (primarily in terms of complex carbohydrates), the reindeer must adapt to seasonal alterations in the carbohydrate component of their diets. It has been earlier reported that lichens (Tolpysheva, 2014; Burkin & Kononenko, 2013) and Embryophyta (Yildirim et al., 2018) consumed by reindeers can contain significant amounts of mycotoxins, which can lead to an imbalance in the ruminal microbiota (Yildirim, 2019). Certain researchers (Sundset et al., 2008, 2009) have considered that anaerobic ruminal microorganisms in reindeer can effectively detoxify the xenobiotics, though earlier research (Kingsbury, 1964) had demonstrated intoxications in reindeer consuming large amounts of lichen.

The adaptation of reindeer to these conditions is associated not only with the genetic potential of the animal itself. The rumen microbiome provides significant assistance in adapting animals to difficult conditions by participating in the digestion of fiber. The ability to digest plant fibers (including cellulose, hemicellulose, and xylans), due to an evolutionally developed symbiosis with the ruminal microbiota, is the unique metabolic feature of ruminants (Koike & Kobayashi, 2009; Ilina, 2012). Taxonomically different members of the gastrointestinal microbial communities are closely interrelated; symbiotic relationships within the ruminal community and its multiplex metabolic activity play a central role in the ruminal functionality, especially in the fermentation of polysaccharides (Creevey et al., 2014). These relationships have been exemplified by the interdependence of the bacterial and archaeal species: certain bacterial species produce hydrogen during the degradation of polysaccharides (Akin, Borneman & Windham, 1988), while certain archaeal species consume this hydrogen for methanogenesis (Janssen & Kirs, 2008). A large part of the lactic acid produced by one bacterial community is further metabolized by another community (Nocek, 1997) for the production of volatile fatty acids, serving as the energetic substrate for the host (Reynolds et al., 1988) and as a buffering agent for the ruminal digesta (Nocek, 1997).

The rumen size of young ruminants are significantly less, in comparison to adult animals. Young ruminants have significantly less relative rumen size, in comparison to adult animals. Certain ruminal functional structures (*e.g*., villi, which increase the absorbing surface) in calves are still underdeveloped (Van Soest, 1982). Age-related changes in the structure and function of the rumen in bovines are also reportedly related to the “maturation” of the ruminal microbiota (Jami et al., 2013).

The variability of habitats and diets can significantly contribute to the investigation of the taxonomic diversity of the ruminal microbiota in reindeer and its metabolic potential (Pope et al., 2012; Salgado-Flores et al., 2016). Hence, the biological and metabolic functions of the ruminal microbiota and their relation to seasonal changes in the diet-and age-related alterations should be further investigated.

The aim of our study was to investigate the taxonomic composition and predicted metabolic pathways of the ruminal microbiota during the winter–spring and summer–autumn seasons in calves and adult reindeer inhabiting the natural pastures of the YNAD to better understanding their adaptation mechanisms to living in arctic areas.

## Materials & methods

### Animals, sampling technique, diets

Ruminal content were sampled during two seasons: summer–autumn (June to September) and winter–spring (December to March) in 14 randomly chosen reindeer (Nenets breed), eight adults aged 2–8 years and six calves aged 4–8 months, during 2017–2018. The samples were taken in the summer–autumn season of 2017 from five adults (AS) and three calves (CS), and in the winter–spring season of 2017–2018 from three adults (AW) and three calves (CW), in their natural habitat in the wooded tundra zone close to the settlement of Kharp (in the YNAD), located in the off spurs of the Arctic Ural (latitude 670N) on the river Sob’, 60 km above the Arctic Circle and 30 km from Labytnanga. All sampled animals were considered healthy, according to their exterior and behavior. The study was approved by L.K. Ernst Federal Science Centre for Animal Husbandry and performed in accordance with Russian Federation ethics legislation with respect to the Russian Federal Law No 498-FZ on Responsible Treatment of Animals.

The ruminal content was sampled manually from the upper part of the ruminal ventral sac by aseptic probes (sample volume: 30–50 ml) in maximally possible aseptic conditions with zond (Medipo-ZT, Czech Republic).

The rumen fluid samples (10 ml) were immediately transferred to aseptic plastic centrifuge tubes, frozen to −20 °C, placed into dry ice, and sent to a molecular genetic laboratory (BIOTROF LTD) for DNA isolation.

The ingredients of the reindeer’s diet were identified by field observations of the pastures (footprints, feces, plant residues). The percentage of each plant eaten was determined by several indirect methods, since the integration of methods provides the most accurate representation in field assessment of diet (Nielsen, 2017). For this, the composition of the entire set of plant species found in the grazing area was taken into account according to the recommendations (Avetisyan & Kosyanenko, 2009). To take into account the percentage of plant species in the grazing area, control plots were allocated: 3 m^2^ in four replicates. The percentage of plants was determined by counting plant specimens at control plots.

Plants consumed by adult animals and calves, in addition to counting plants in the pasture, were also determined by remote observation of feeding (Tollit, 2006). In addition, the species composition of the remains of the eaten plants was assessed.

The summer–autumn vegetation supposedly consumed by the animals included lichens from the genera *Cladonia* (5% of total vegetation) and *Nephroma* (5%); woody plants were represented by *Bеtula pendula*, 5%; brushwoods by *Sаlix borеalis*, 5%; *Vaccinium uliginosum*, 10%, and *B. nаna*, 25%; fruticuloses by *S. polaris*, 15%; perennial grasses (30% of total) by *Poa arctica*, *Eriophorum vaginatum*, *Calamagrostis epigeios*, *Tanacetum vulgáre*, *Galium boreale*, and *Alopecurus pratensis*. The winter–spring diets included lichens (*Cladonia* sp. and *Nephroma* sp., 60%), *Vaccinium uliginosum* (10%), *B. nаna* (10%), and grassland litter and sub-nival greenery (20%). The taxonomy of the vegetation was identified using the botanical guides by Stankov & Taliev (1949) for higher plants and Oksner (1974) for lichens. No additional feedstuffs were fed to the reindeer in both seasons.

### Chemical analysis of dietary vegetation

Poly-component samples of the vegetation, identical to the averaged composition of winter–spring and summer–autumn diets of the reindeer, were taken in three replicates each. Fresh plants were weighed and dried at 80 °C for 72 hours. In the dried samples, the percentages of dry matter (by drying at 105 °C), crude protein (by Kjeldahl method), crude fat (by Soxhlet extraction), soluble carbohydrates (by colorimetric Anthrone method), and crude fiber (by the method of Henneberg and Stohmann) were determined; the content of metabolizable energy was also calculated, according to the procedures by Kalashnikov & Kleymenov (2003) and Kharitonov, Jabarov & Grigoryeva (2012).

### DNA isolation

Total bacterial genomic DNA was extracted from all the collected ruminal content samples and used as template in the 16S rRNA gene PCR amplification. Total DNA from the samples of ruminal content was isolated with a Genomic DNA Purification Kit (Thermo Fisher Scientific), as previously described in our study (Ilina et al., 2021). The procedure protocol (https://assets.thermofisher.com/TFS-Assets/LSG/manuals/MAN0012656_Genomic_DNA_Purification_UG.pdf) was based on the use of lysis solution, chloroform, NaCl solution and ethanol.

### 16S rRNA amplicon library preparation

The analysis of ruminal microbiota was performed by the Next Generation Sequencing (NGS) using the MiSeq (Illumina, San Diego, CA, USA) platform, with primers for the V3–V4 region of 16S rRNA (Klindworth et al., 2013; Johnston et al., 2017): Upstream primer 5′ TCGTCGGCAGCGTCAGATGTGTATAAGAGACAGCCTACGGGNGGCWGCAG; downstream primer 5′ GTCTCGTGGGCTCGGAGATGTGTATAAGAGACAGGACTACHVGGGTATCTAATCC.

The following reagent kits (Illumina, San Diego, CA, USA) were used: Nextera® XT IndexKit for preparation to sequencing, Agencourt AMPure XP for purification of PCR products, and MiSeq® Reagent Kit v2 (500 cycle) for sequencing. All preparations were made according to the Illumina protocol (https://support.illumina.com/content/dam/illumina-support/documents/documentation/chemistry_documentation/16s/16s-metagenomic-library-prep-guide-15044223-b.pdf). The pool concentration was 4 nm. The maximal length of the sequences obtained was 2 × 250 bp.

### Sequence processing and analysis

Bioinformatic analysis of the data was performed using the QIIME 2 ver. 2020.11 software (Bolyen et al., 2019). We have imported the sequences into QIIME 2 format. Demultiplexed sequences contained 6,232 to 127,969 reads, averaging 46,036.5 reads per sample. The Quality Score median of reads for forward and backward reads was 37.3 and 36.2 respectively (Phred 33). After the import of the sequences into QIIME 2, the paired lines of the reads were equaled and the sequences were filtered, according to their quality, using standard filtration parameters. Noise sequences were filtered using the DADA2 method, with the quality of reads less than two (truncQ = 2), reads shorter than 250 bp (truncLEN = 250) were discarded, reads with the number of expected errors more than two were also discarded (https://benjjneb.github.io/dada2/; Callahan et al., 2016). The minimum, maximum and average lengths of the combined pair-terminal sequences were 275, 467, and 459 bp, respectively. For the *de novo* construction of the phylogeny, multiple-sequence alignment *via* MAFFT was performed (Janssen & Kirs, 2008), with subsequent masking of non-descriptive sequences. We used the taxonomy of the Silva reference database (https://www.arb-silva.de/documentation/release-138/).

For all the data of the table with amplicon sequence variant (ASV) alpha diversity indices were calculated using plugins of the QIIME 2 ver. 2020.11. A graph of the dependence of the number of ASVs on the number of readings were also built. The average number of ASVs per sample was 8,645.5, the minimum and maximum numbers were 2,181.0 and 17,679.0 respectively. On the basis of the table of the operational taxonomic units (OTUs), the indices of alpha-biodiversity were calculated and the graph of the dependence of number of OTUs on the number of reads was plotted using plug-in software from the QIIME 2 ver. 2020.11 package. The statistical analysis of the indices did not involve any additional transformations.

The network of microbial associations was built using the method of simulation modeling SPIEC-EASI, based on the data of 16S rRNA sequences obtained from all samples. The resulting network was scale-free, *p* = 0.997 (according to the Kolmogorov–Smirnov test), and featured a relatively small number of strongly inter-related vertices. After importing the ASV table and metadata into the R, the NA sequences at all taxonomic levels were deleted, the remaining 2,757 taxonomic units were filtered according to the criterion of minimum occurrence. With these data, the SPIEC-EASI pipeline was launched, the sparse inverse covariance matrix was calculated using the of MB method (Meinshausen & Bühlmann, 2006). With the target threshold of 0.05, the stability of the network was 0.0498, which indicates that the parameters were correctly selected for building the network. Clusters with dominating bacteria were identified using the igraph package for R (https://igraph.org/r/). The final style of the network was formed by means of Cytoscape (Shannon et al., 2003). On the basis of the 16S rRNA sequencing, the networks of microbial associations for all sample groups were plotted using sparse inverse covariance estimation for ecological association inference (SPIEC-EASI) (Kurtz et al., 2015), the method of Meinshausen & Bühlmann (2006), and the R software (https://github.com/zdk123/SpiecEasi). Clusters with dominating bacteria were identified using the igraph package for R (https://github.com/igraph/rigraph).

### Functional prediction of the metagenome

The reconstruction and functional prediction of the metabolic pathways of the metagenome, gene families, and enzymes were performed using the PICRUSt2 (v.2.3.0) software, with the recommended scenario and settings (Douglas, Maffei & Zaneveld, 2020). The amplicon sequence variants (ASV) were transferred to the standard pipeline for Picrust2 (v.2.3.0) (Louca & Doebeli, 2018). The average NSTI value was calculated for each sample. ASV sequences with NSTI values above two were excluded from the prediction. Based on the prediction of the Enzyme Classification numbers, we obtained a prediction of MetaCyc metabolic pathways. After that, the abundance of ASV in the profiles of metabolic pathways was assessed using the MetaCyc database (https://metacyc.org/). For the analysis of metabolic pathways and enzymes, the MetaCyc database (https://metacyc.org/) and comparison of the abundance of amplicon sequence variants were used.

### Mathematical and statistical analysis

Statistical processing was carried out using Microsoft Excel XP/2003 and R-Studio (v. 1.1.453) software. The Shapiro-Wilk test was used to assess the distribution pattern in the aggregate based on alpha diversity and pasture diets. Comparisons of two groups from populations with normal distribution were performed using Student’s t-test. To analyze sample data from populations that differ from the normal distribution, the Mann-Whitney test was used. Subsequent intergroup comparisons of predicted metabolic pathways and microbial community were performed using Tukey’s test. The analysis of the influence of factors for season and age was carried out using the ANOVA method. Differences were considered significant at *p* < 0.05. Numerical data are presented as means (M) and standard errors of means (±SEM). The data obtained were analyzed using the ANOVA method and Microsoft Excel XP/2003 and R-Studio (v. 1.1.453; https://rstudio.com) software. The normality of data distribution and homogeneity of variance were assessed using the Shapiro–Wilk and Levene’s tests, respectively. The effect of multiple comparisons was corrected using Tukey’s HSD test from the relative R package (https://www.rdocumentation.org/packages/stats/versions/3.6.1/topics/TukeyHSD). Differences were considered significant at *р* < 0.05. The data are presented as mean values (M) and standard errors of the means (±SEM). The indices of biodiversity of the ruminal microbiota (Shannon’s H, Simpson’s D) were calculated with the control of two consistency criteria.

### Accession numbers

The 16S rRNA gene sequences were deposited in the National Center for Biotechnology Information (NCBI) Sequence Read Archive (SRA) under BioProject, with accession number PRJNA576999.

## Results

### The composition of diets

The nutritive parameters of summer–autumn diets (CS and AS) and winter–spring diets (CW and AW), as presented in Table 1, significantly differed. The averaged (between ages) winter–spring diets contained a significantly higher amount of crude fiber (by 22.4%, *p* < 0.05) and lesser amounts of crude protein (by 74.6%, *p* < 0.001) and crude fat (by 54.4%, *p* < 0.05), in comparison to summer–autumn diets.

**Table 1:** Averaged biochemical composition of summer–autumn (CS and AS groups) and winter–spring (CW and AW groups) pasture diets of calves and adult reindeer.

No.	Parameter	Average values for the pairs of the seasonal groups	
		S (CS, AS)	W (CW, AW)
1	Dry matter (DM), %	76.7 ± 1.60	76.5 ± 2.72
2	Crude fiber (g/kg of DM)	142.8 ± 6.31	174.8 ± 7.20*
3	Soluble carbohydrates (sugars) (g/kg of DM)	20.8 ± 0.80	32.4 ± 1.41*
4	Crude fat (g/kg of DM)	16.9 ± 0.61	7.70 ± 1.89*
5	Crude protein (g/kg of DM)	95.9 ± 4.75	24.2 ± 1.74**
6	Crude ash (g/kg of DM)	36.4 ± 1.12	34.8 ± 1.40
7	Metabolizable energy (MJ/kg of DM)	9.44 ± 0.45	8.92 ± 0.42

**Note:**

Seasonal differences were significant, according to the Student’s T-test, at: *р ≤ 0.05, **р ≤ 0.001.

**Figure 1:**
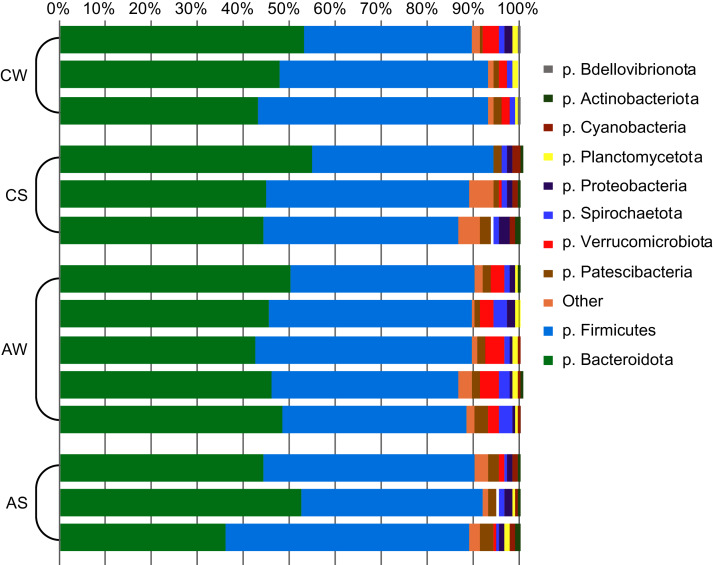
Composition of ruminal microbiota in reindeer: Bacterial phyla identified by NGS of amplicons of 16S rRNA. CW, calves in winter–spring season; CS, calves in summer–autumn season; AW, adults in winter–spring season; AS, adults in summer–autumn season.

In total, 22 bacterial phyla were identified (Fig. 1), with phyla Bacteroidetes and Firmicutes dominating (up to 48.1% ± 4.30% and 46.1% ± 4.80%, respectively), while two archaeal phyla were minor communities (no more than 0.54% ± 0.14% in total).

The assortativity coefficient (*i.e*., Pearson’s correlation coefficient between the degrees, or edge numbers, of the adjacent vertices) for the entire network was 0.066. This coefficient, for different taxonomic levels, differed insignificantly: r = 0.014 between phyla, r = −0.011 for classes, r = 0.015 for orders, r = 0.001 for families, and r = 0.020 for genera. These data indicate that the degree of assortativity of the entire network was close to zero and that the vertices with high degree (so-called “stars”) tended to be related with other high-degree vertices directly (a feature of assortative networks) and by chains of low-degree vertices (a feature of disassortative networks).

**Figure 2:**
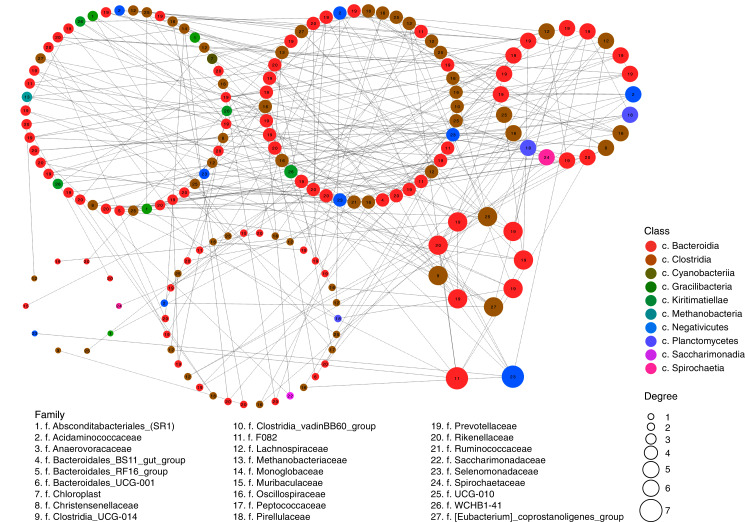
Network of possible interrelations between different classes and families of prokaryotic microorganisms in the ruminal community of the reindeer. Network was plotted using the SPIEC-EASI method (Kurtz et al., 2015), the method of Meinshausen & Bühlmann (2006), and the R software (https://github.com/zdk123/SpiecEasi). Clusters with dominating bacteria were identified using the igraph package for R (https://github.com/igraph/rigraph). Circles represent the vertices (taxonomic units), while lines represent the edges between them. The diameters of the circles represent the numbers of edges (degree), the color of the circle reflects the class, and figures within the circles reflect the family.

These inter-relations are exemplified in Fig. 2, at the level of classes and families. The network had 202 positively correlated edges and 77 negatively correlated edges. Concentrator classes (with maximal edge numbers) were identified using the greedy clustering algorithm (Clauset, Newman & Moore, 2004). The highest degree (7) was found for classes Bacteroidia (phylum Bacteroidetes) and Negativicutes (phylum Firmicutes); while class Clostridia (phylum Firmicutes) had six edges. The average degree for all vertices of the network was 3.38.

**Figure 3:**
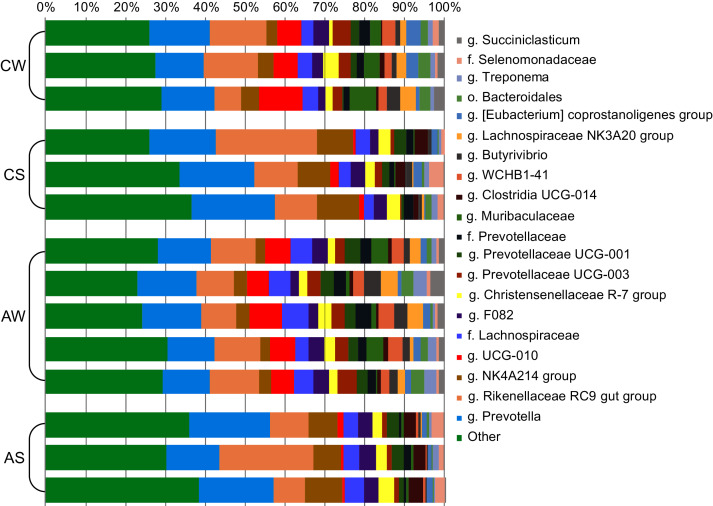
Composition of ruminal microbiota in reindeer: Bacterial genera identified by the sequencing of amplicons of 16S rRNA. CW, calves in winter–spring season; CS, calves in summer–autumn season; AW, adults in winter–spring season; AS, adults in summer–autumn season.

At the genus level (Fig. 3), the most abundant in the rumen were bacterial genera from phylum Bacteroidetes: Prevotella (family Prevotellaceae, from 13.26% ± 0.752% to 18.75% ± 1.497%) and Rikenellaceae RC9 (family Rikenellaceae, from 10.68% ± 0.794% to 15.74% ± 5.965%); and phylum Firmicutes: Oscillospiraceae NK4A214 group (family Oscillospiraceae, from 3.70% ± 0.626% to 9.08% ± 0.931%) and Ruminococcaceae UCG-010 group (family Ruminococcaceae, from 1.36% ± 0.534% to 7.72% ± 1.898%).

### Comparison of composition of microbiota between groups

#### Alpha-diversity analysis

The parameters of alpha-biodiversity in different samples were calculated on the basis of the results of sequencing of 16S rRNA amplicons, as presented in Table 2. Both Shannon’s and Simpson’s diversity indices did not significantly differ (*p >* 0.05) between the samples.

**Table 2:** Shannon’s and Simpson’s indices of alpha-biodiversity for the ruminal microbiota in reindeer (calculated with plugins for the QIIME 2 ver. 2020.11 software package).

Treatment	Shannon’s index	Simpson’s index
CS	7.20 ± 0.316^a^	0.991 ± 0.0023^b^
CW	7.13 ± 0.490^a^	0.989 ± 0.0039^b^
AS	8.43 ± 0.152^a^	0.996 ± 0.0009^b^
AW	7.867 ± 0.246^a^	0.995 ± 0.0010^b^

**Note:**

a, b–Figures in lines with different superscripts differed insignificantly (*р* ≥ 0.05).

#### Similarity analysis

**Figure 4:**
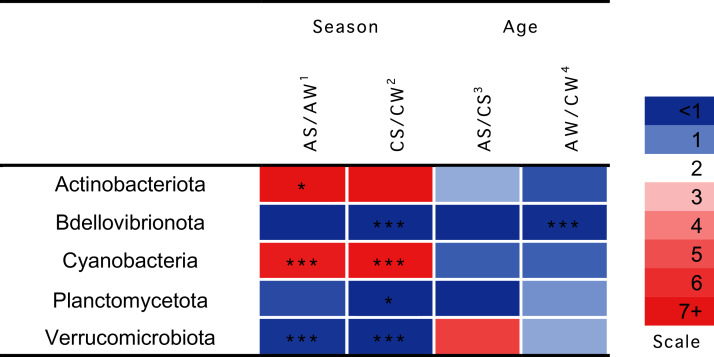
Significant season-and age-related differences between the treatments of reindeer in the composition of ruminal microbiota (phylum level), identified by sequencing of 16S rRNA amplicons. CW, calves in winter–spring season; CS, calves in summer–autumn season; AW, adults in winter–spring season; AS, adults in summer–autumn season. Colors of the cells represent the multiplicity factor (see the scale in the left part); *p < 0.05, **p < 0.01, ***p < 0.005.

Significant seasonal changes in the composition of ruminal microbiota (Fig. 4) involved bacterial phyla Actinobacteria, Bdellovibrionota, Planctomycetes, and Verrucomicrobia and Cyanobacteria (*p* < 0.05), the largest being the changes in Actinobacteria and Cyanobacteria. The increase in the concentration of Actinobacteriota in the summer–autumn season, compared to the winter–spring season, in adults was 19.3-fold (*p* = 0.02); while that in Cyanobacteria was up to seven-fold in calves and adults (*p* = 0.002). Significant age-related changes were found only for Bdellovibrionota in the winter–spring season (*p* = 0.0036).

**Figure 5:**
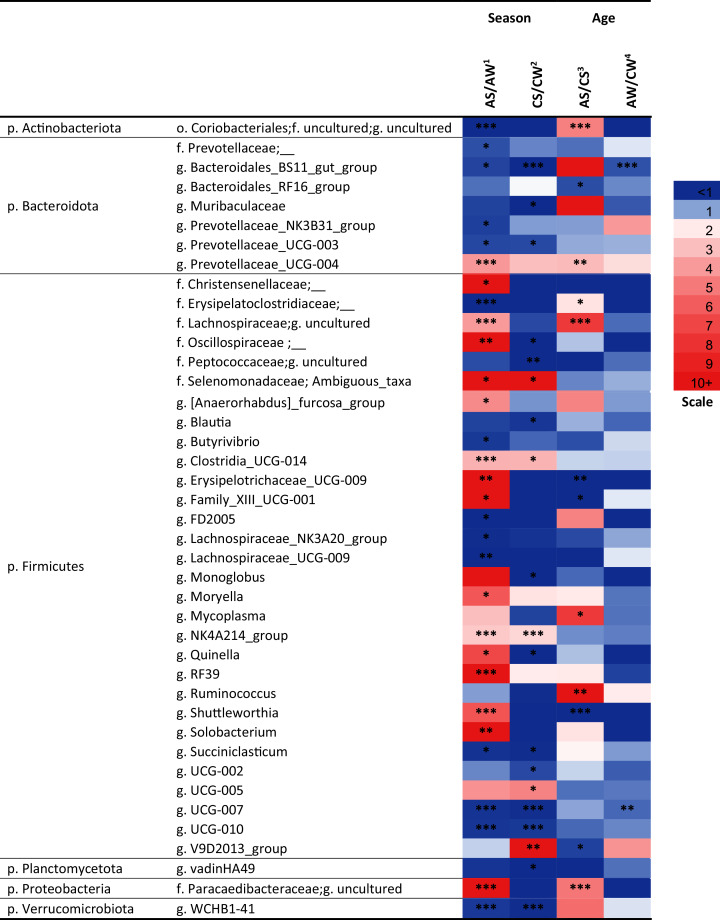
Significant season-and age-related differences between the treatments of reindeer in the composition of ruminal microbiota (genus level), identified by sequencing of 16S rRNA amplicons. CW, calves in winter–spring season; CS, calves in summer–autumn season; AW, adults in winter–spring season; AS, adults in summer-autumn season. Colors of the cells represent the multiplicity factor (see the scale in the left part); *p < 0.05, **p < 0.01, ***p < 0.005.

Over a half of all bacterial genera identified as significantly different (*p* < 0.05) between the samples (*e.g*., the NK3B31 group, UCG-003, UCG-004, NK4A214 group, RF39, vadinHA49, WCHB1-41, and so on) were uncultivated (Fig. 5).

At the genus level, the seasonal changes were also more substantial (especially in adults), compared to age-related changes (see Fig. 5). Significant differences (*p* < 0.05) were found between the concentrations of the genera from Firmicutes phylum. In the AS group, a significant increase (up to 49.6-fold, *p* < 0.05) in the total concentration of 14 uncultivated Firmicutes genera, in comparison to AW, was found. This group included uncultivated genera from order Christensenellaceae, families Lachnospiraceae and Oscillospiraceae, Ambiguous taxa (family Selenomonadaceae), Erysipelotrichaceae UCG-009, *Moryella*, *Shuttleworthia*, *Solobacterium*, and so on (see Fig. 5). On the contrary, the total concentrations of eight other Firmicutes genera—*Butyrivibrio*, FD2005, NK3A20 group, UCG-009 (family Lachnospiraceae), UCG-007, UCG-010, and so on—in AS was up to 27.8-fold lower, in comparison to AW (*p* < 0.05). Concentrations of certain genera (uncultivated genera of families Erysipelotrichaceae, Clostridiaceae and Lachnospiraceae, UCG-009, UCG-001, *Shuttleworthia*) significantly varied in a two-way manner between the ages in the summer–autumn season (*i.e*., in AS compared to CS; *p* < 0.05).

Among the genera of Bacteroidetes, the most variable was an uncultivated genus “BS11 gut group”. Significant decreases in the concentrations of this taxon in the winter–spring season, compared to the summer–autumn season, were found in calves (from 2.169 ± 0.22 in CS to 0 in CW; *р* = 0.00001) and adults (from 0.91 ± 0.14 in AS to 0.26 ± 0.29 in AW, *р* = 0.04). A significant decrease in its concentration in adults (AW), compared to calves (CW), in the winter–spring season was also found (*p* = 0.0006).

Significant differences between the samples (*p* < 0.05) were also found for certain bacterial genera from Actinobacteria, Planctomycetes, and Verrucomicrobia, as well as from phylum Proteobacteria; for example, the concentration of an uncultivated genus from order Coriobacteriales (Actinobacteria) significantly differed between AS and AW and between AS and CS samples(*p* < 0.005).

### Predicted metabolic pathways of ruminal microbiota

**Figure 6:**
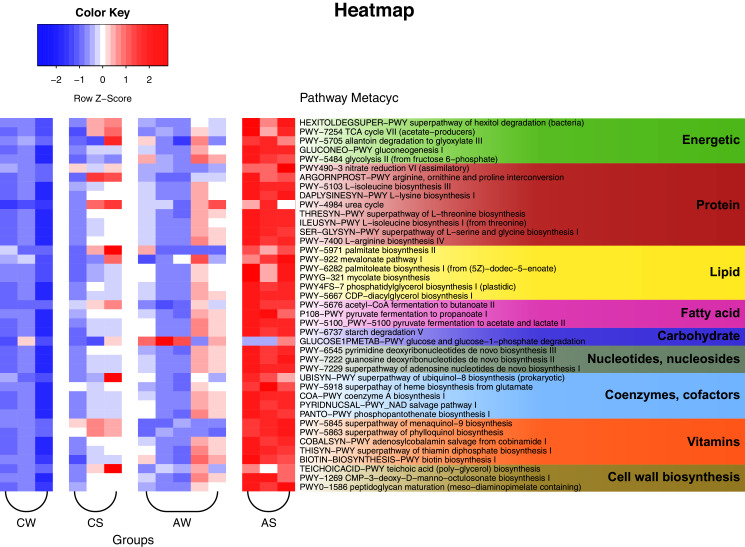
Heat map of the predicted metabolic pathways of ruminal microbiota in reindeer, plotted using the PICRUSt2 (v.2.3.0) software and the MetaCyc database (https://metacyc.org/). CW, calves in winter–spring season; CS, calves in summer–autumn season; AW, adults in winter–spring season; AS, adults in summer–autumn season.

The PICRUSt2 data and analysis with the use of KEGG (Kyoto Encyclopedia of Genes and Genomes) identified a total of 257 predicted metabolic pathways for the ruminal microbiota in reindeer (Fig. 6). Among these pathways, 25 could be nominally related to the metabolism of carbohydrates and energy (*e.g*., Krebs cycle, glycolysis, degradation of hexitols to phosphoenolpyruvate, and allantoin to glyoxylate); 47 to the metabolism of protein (*e.g*., biosynthesis of amino acids, allantoin, and transformation of nitrous compounds); 19 to the metabolism of lipids (*e.g*., mevalonate pathway, biosynthesis of lipids, mycolic acids, oleate, palmitate, phosphatidylglycerol, and so on); 13 to the synthesis of volatile fatty acids (VFAs), including lactate (5 pathways); 11 to the degradation of complex polysaccharides, including glucose; 33 to the synthesis of nucleic acids, nucleotides, and nucleosides; 20 to the synthesis of cofactors and coenzymes (*e.g*., acetyl-CoA, ubiquinol, NAD, and phosphopantothenate); 23 to the synthesis of vitamins (*e.g*., menaquinol, adenosylcobalamin, thiamin diphosphate, biotin, and tetrahydrofolate); and 10 to the synthesis of structural elements of cell walls (*e.g*., murein, teichoic acids, and CMP-3-deoxy-D-manno-octulosonate). In addition, certain minor pathways were identified as being related to pathogenesis, methanogenesis, biosynthesis of antimicrobials, formation of biofilms, sulfur oxidation, Calvin cycle, biosynthesis of butanediol, degradation of neuromediators, and so on.

### Comparison of the metabolic pathways of microbiota between groups

**Figure 7:**
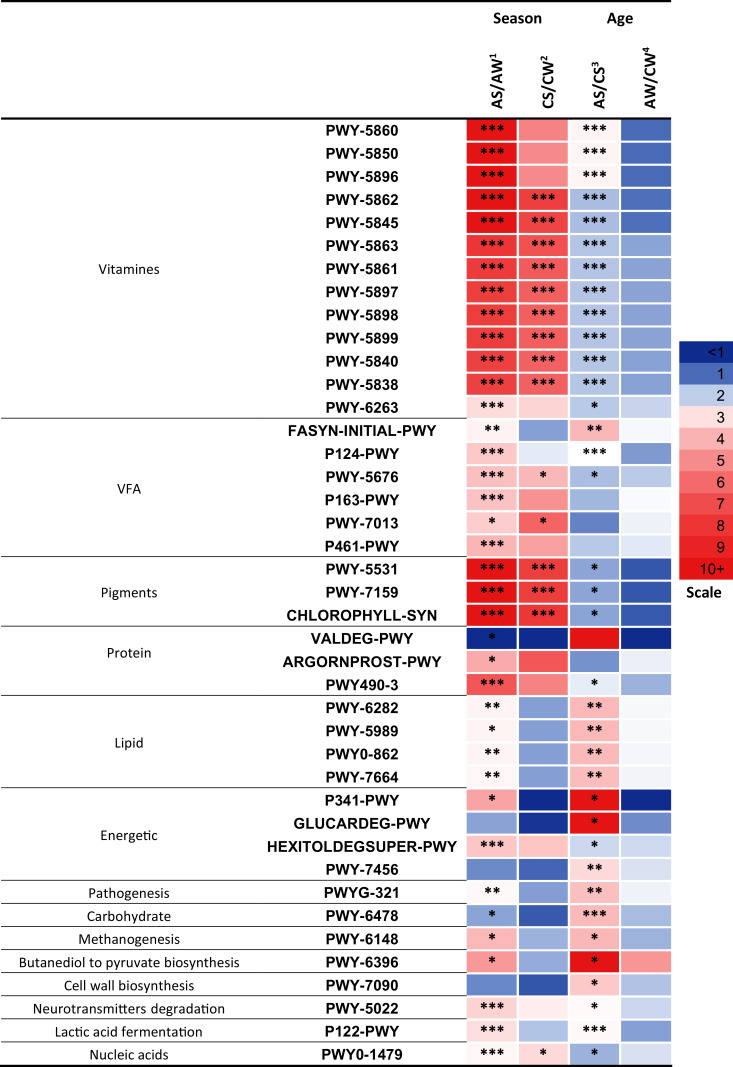
Significant season-and age-related differences between the treatments of reindeer in the metabolic pathways of ruminal microbiota identified by sequencing of 16S rRNA amplicons. CW, calves in winter–spring season; CS, calves in summer–autumn season; AW, adults in winter–spring season; AS, adults in summer–autumn season. Colors of the cells represent the multiplicity factor (see the scale in the left part); *p < 0.05, **p < 0.01, ***p < 0.005.

Significant differences (*p* < 0.05) between the samples were found in 41 metabolic pathways (Fig. 7) related to the synthesis of vitamins, VFA, pigments; the metabolism of proteins, lipids, and energy; pathogenesis, degradation of carbohydrates, methanogenesis, transformation of butanediol to pyruvate, cell wall biosynthesis, degradation of neurotransmitters, lactic acid fermentation, and biosynthesis of nucleic acids.

It should be emphasized that a large part of the pathways that differed between the groups (13 pathways, *p* < 0.05) was related to the biosynthesis of different homologues of vitamin K, resulting in the synthesis of phylloquinone, menaquinols with various number of isoprene residues, and so on: PWY-5862-superpathway of demethylmenaquinol-9 biosynthesis, PWY-5845-superpathway of menaquinol-9 biosynthesis, PWY-5837-1,4-dihydroxy-2-naphthoate biosynthesisI, PWY-5863-superpathway of phylloquinol biosynthesis, PWY-5861-superpathway of demethylmenaquinol-8 biosynthesis, PWY-5897-superpathway of menaquinol-11 biosynthesis, PWY-5898-superpathway of menaquinol-12 biosynthesis, PWY-5899-superpathway of menaquinol-13 biosynthesis, PWY-5840-superpathway of menaquinol-7 biosynthesis, PWY-5838-superpathway of menaquinol-8 biosynthesis I, and so on. These 13 pathways differed between the AS and AW (up to 13.3-fold higher in AS, *p* < 0.005) and between AS and CS (up to 2.6-fold higher in AS, *p* < 0.05). Only nine pathways differed between CS and CW (up to 7.6-fold higher in CS, *p* < 0.005; Fig. 7). These pathways tended to be significantly more active in the summer–autumn season, compared to winter-spring, and in adult reindeer, in comparison to calves, during the summer–autumn season.

**Figure 8:**
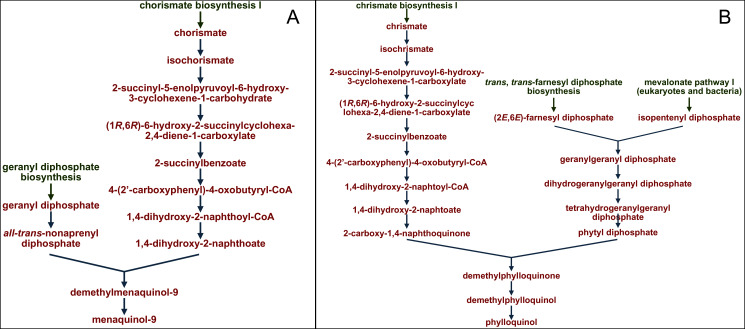
Reconstruction and visualization of predicted metabolic pathways. (A) PWY-5845 (superpathway of menaquinol-9 biosynthesis) and (B) PWY-5863 (superpathway of phylloquinol biosynthesis) in ruminal microbiota of reindeer using the MetaCyc database (https://metacyc.org/).

The profiles of certain pathways that significantly differed between samples were reconstructed and visualized with use of the MetaCyc database (https://metacyc.org/), as exemplified in Fig. 8 for the pathways PWY-5845 and PWY-5863 (involved in the biosynthesis of two vitamin K homologues).

We established that the main precursor in the pathways of microbial biosynthesis of most vitamin K homologues was chorismic acid (as exampled on Fig. 8).

The significant seasonal increase (*p* < 0.005) in the activity of the biosynthesis of a chlorophyll precursor (chlorophyllide) in the summer–autumn season was also clear, both in calves (up to 5.1-fold) and adults (up to 13.5-fold).

It is interesting to note that the activities of the predicted metabolic pathways related to the synthesis of VFA, metabolism of protein, lipids, and energy, pathogenesis, degradation of carbohydrates, methanogenesis, transformation of butanediol to pyruvate, degradation of neurotransmitters, and lactic acid fermentation differed primarily between the AS and AW and between AS and CS (*p* < 0.05) that is, in relation to season in adults and in relation to age in the summer–autumn season (see Fig. 7) while the difference between AW and CW was insignificant (*p* > 0.05) and difference between CS and CW was negligible.

## Discussion

### Biochemical composition of reindeer’ diets

Our data evidenced that the nutritive value of dietary ingredients in the winter–spring season was lower than that in the summer–autumn season. The increased content of fiber in the winter–spring diet indicated the lower level of its digestibility by reindeer and the necessity of the adaptation to the composition of ruminal microbiota with regard to this and other seasonal dietary changes. On the other hand, the winter–spring diet contained a significantly lesser amount of crude protein.The low content of crude protein in lichens (including *Nephroma* genus) in winter, despite the presence of nitrogen-fixing Cyanobacteria, has been previously reported (Pulliainen, 1971); certain authors have also reported the negative nitrogen balance and body weight losses in reindeer having diets rich in lichens (Staaland, Jacobsen & White, 1984; Aagnes & Mathiesen, 1995; Oksendal, 1994). In addition, the winter diet can negatively affect the biosynthesis of microbial protein by the ruminal microbiota. The significantly decreased fat content in the winter diet was also unfavorable for the reindeer, as fats are more effective energy sources for ATP synthesis, compared to VFA or protein catabolism (Palmquist, 1994). Generally, our data supported the suggestion regarding the negative impact of winter–spring diets on the nutritional status in reindeer, compared to summer–autumn diets.

### Dominant microbial taxa in the rumen

The sequencing of microbial 16S rRNA resulted in numbers of OTUs ranging from 746 to 7,765 per sample, evidencing a high level of complexity of ruminal microbiota in reindeer.

In all samples, a total of 22 bacterial phyla were identified, the dominant ones being phylum Bacteroidetes and Firmicutes. These taxa have been previously reported to dominate the ruminal community in all studied reindeer sub-species (Pope et al., 2012; Salgado-Flores et al., 2016) and, hence, can be regarded as the dominant of the ruminal bacterial community. Interestingly, these phyla have been also detected as dominant in the intestinal microbial communities of many mammal species (Ley et al., 2008; Shanks et al., 2011), indicating their important functional and ecologic roles in the digestive tract of animals. The important function of Firmicutes is the degradation of complex polysaccharides, with subsequent synthesis of VFA, which is degradable for ruminants; while Bacteroidetes are primarily degraders of carbohydrates, fats, and proteins (Jami et al., 2013).

For prediction of the possible interactions between different taxa in the ruminal microbiota of reindeer, a network of microbial associations was built using the SPIEC-EASI method of simulation modeling was build. The bacteria with the maximal numbers of edges (concentrators) were found to belong to the class Bacteroidia (phylum Bacteroidetes) and classes Negativicutes and Clostridia (phylum Firmicutes). These taxa are evidently responsible for the largest part of the ruminal metabolism; primarily in the metabolism of complex carbohydrates, protein, monosaccharides, and organic acids, as these substances are the main components of their diets and are important intermediates in the metabolism of energy, as has been shown in our analysis of reindeer dietary composition and earlier data on the nutrition of reindeer (Pulliainen, 1971; Nilsson et al., 2006; Mathiesen et al., 2006). It is safe to assume that these taxon-concentrators play a key role in the formation of the sustainable core of the ruminal microbiota in reindeer.

The taxonomic analysis at genus level revealed that the bacterial genera Prevotella and Rikenellaceae RC9 (phylum Bacteroidetes) and Oscillospiraceae NK4A214 group and Ruminococcaceae UCG-010 group (phylum Firmicutes) were most abundant. The prevalence of Prevotella has also been observed in the ruminal microbiota of certain species of deer (Li et al., 2013; Qian et al., 2017), including *Cervus canadensis* (Gruninger et al., 2014), as well as other ruminants, including bovines (Jami & Mizrahi, 2012; Henderson et al., 2015; Xin et al., 2019). The dominance of Prevotella in the ruminal microbiota is not surprising, as many species of this genus are polysaccharide degraders (Dehority, 1967; Coen & Dehority, 1970); for example, Gasparic et al. (1995) have reported the presence of genes related to the activities of xylanase and endoglucanase and to the synthesis of lichenin-degrading enzymes in the genome of *P*. *ruminicola*. The presence of *Prevotella* species in the rumen has been suggested to improve the adaptation of ruminants to diets rich in poorly digestible ingredients (Culberson, 1969; Van Soest, 1982; Scheller & Ulvskov, 2010), the main products of *Prevotella* metabolism being succinate and acetate (Bryant et al., 1958). The uncultivated species from the Ruminococcaceae UCG-010 group have been also reported to be related to the degradation of poorly digestible polysaccharides, including cellulose (Opdahl, Gonda & St-Pierre, 2018).

### Composition of ruminal microbiota: effects of season and age

In our study, the Shannon’s and Simpson’s indices of alpha-biodiversity of the ruminal microbiota (Reese & Dunn, 2018) did not significantly differ between the samples. The percentages of the dominant taxa (phyla Bacteroidetes and Firmicutes) were also constant. These results are interesting, as no expectable changes (in both directions) were found in the diversity and percentages of the dominating taxa in the winter–spring season, as a result of the effects of certain unfavorable external factors on the host and/or the microbiota. These factors include the abrupt decrease in diet quality, cold stresses, and deficits of nutrients and energy. An analysis of data from 32 ruminant species in different regions of the World (Henderson et al., 2015) has revealed diet to be the main factor affecting the composition of the ruminal microbiota—the diet has been found to be more influential than all other factors, including host genotype and individual physical characteristics (*e.g*., immune status). It has been also established that nutrition involving the use of concentrates can result in serious dysbiosis of the ruminal microbiota and cause severe metabolic disorders in cattle (Nocek, 1997; Ilina, 2012).

Our data evidenced that the composition and biodiversity of the dominant ruminal community in reindeer are stable. This fact probably indicates that the physiology of reindeer—including the function of the ruminal microbiota—is far less affected by anthropogenic factors, in comparison to cattle; for example, Aagnes & Mathiesen (1995) have reported a lower taxonomic diversity of ruminal community in reindeer fed exclusively on natural pastures, in comparison to cattle additionally fed alfalfa hay and concentrates. Our data are in agreement with previous reports (Weimer, 2015) on the stability of the gastrointestinal microbiome, being unaffected by external factors. The absence of differences between the ruminal communities (at the level of dominating phyla) between calves and adults in our study can be related to the age of the sampled calves: The ruminal microbiota in calves at 4–8 months of age was evidently matured and similar to that in adults, while the seasonal stability of this community probably reflects the evolutional adaptation of reindeer to the natural sequences of seasonal changes in diets.

Despite the stability of this ruminal community, concentrations of certain minor communities (phyla and genera) significantly differed between the groups.

The seasonal factor primarily affected the concentrations of Actinobacteria and Cyanobacteria: The summer-related increases in the concentrations of these taxa were expected, as these microorganisms are typical of the epiphyte and endophyte microbiota of Embryophyta (Ariosa et al., 2004; Nair & Padmavathy, 2014; Singh & Dubey, 2015, 2018), which appeared in the summer diets of reindeer. Meanwhile, Actinobacteria species are popular symbionts of the digestive system in eukaryotes (Lewin et al., 2016); their cellulolytic enzymes promote the degradation of cellulose (Berlemont & Martiny, 2013). Cyanobacteria species feature nitrogenase activity and are capable of synthesizing cyanotoxins (microcystins) (Fastner et al., 2002).

The factor of age in our study was found only for the Bdellovibrionota during the winter–spring season. These microorganisms, which are permanent inhabitants of the digestive system (Iebba et al., 2013), are mostly bacteriovores (*e.g*., *Bdellovibrio bacteriovorus*), which use other Gram-negative species as a substrate for growth and reproduction and, therefore, may act as natural balancers of their populations (Rendulic et al., 2004; Dwidar, Monnappa & Mitchell, 2012). In our opinion, the increase in the concentration of Bdellovibrionota with age is beneficial for the reindeer, as the dysbioses induced by different diseases are commonly accompanied by decreases in the population of bacteriovores, including *B. bacteriovorus* (Iebba et al., 2013); to the contrary, experimental oral administration of *Bdellovibrio* spp. in chickens has been reported to reduce the populations of undesirable Gram-negative species in the intestinal microbiota (*e.g*., Salmonellas) and soften the inflammation of the intestinal mucosa (Atterbury et al., 2011). Therefore, bacteriovorism can be regarded as an important natural mechanism for the regulation of the concentrations of bacterial communities (Chen & Williams, 2012).

The differences between the groups in the composition of ruminal microbiota at the genus level in our study were caused more by season than by age (similarly to the phylum level); the most significant changes were found in adults. It is interesting to note that most of the affected genera from phylum Firmicutes were those that ferment plant polysaccharides to organic acids, including VFA: uncultivated genera from families Lachnospiraceae (Downes et al., 2002; Cepeljnik et al., 2003; Sizova et al., 2014), Oscillospiraceae (Pascual et al., 2020), Selenomonadaceae (Tarakanov, 2006), genera *Shuttleworthia* (Downes et al., 2002), *Butyrivibrio* (Fukuda et al., 2005), and so on. These genera are common inhabitants of the rumen in different ruminants (Zhu et al., 2018; Grum et al., 2020; Laishev et al., 2020). Earlier, Hu et al. (2018) reported seasonal changes in the population of phylum Firmicutes in the intestinal microbiota of forest musk deer (*Moschus berezovskii*), including family Lachnospiraceae. Salgado-Flores et al. (2016) have demonstrated a decrease in certain Firmicutes taxa in the ruminal microbiota of reindeer fed with lichens, in comparison to those fed with standard pelleted compound feeds.

The seasonal changes in the concentrations of the genera from the other dominating phyla, Bacteroidetes, in our study were also more evident than age related changes; for example, the increases in the concentration of uncultivated genus “BS11 gut group” in adults and in calves were found in the winter–spring season, in comparison to summer–autumn. This shift, together with the shifts in the concentrations of other taxa, may indicate the evolution of dysbiotic conditions in the winter–spring season; for example, it is well-known that certain nutritional imbalances in cattle can lead to an increase in the ruminal population of Bacteroidetes species, resulting in the active synthesis of lactate as the intermediate of the fermentation of starch and monosaccharides. The accumulation of lactate, in turn, impairs the ability of the ruminal microbiota to effectively maintain the acid–base equilibrium in ruminal digesta, resulting in the accumulation of protons in the lumen and a decrease in ruminal pH below the physiologically normal range; this disorder is known as ruminal (or lactate) acidosis (Zebeli et al., 2012; Krause & Oetzel, 2006). It is interesting to note that gastrointestinal diseases and necrobacteriosis (purulent necrotic lesions of the distal segments of the limbs) are among the main factors limiting the growth of reindeer populations: In 2009, ca. 65,000 of domesticated reindeer died of necrobacteriosis (Zabrodin et al., 2019). The causative agent of necrobacteriosis is the highly virulent pathogen *Fusobacterium necrophorum*, which is known to be resistant to low pH conditions and to use lactate as a substrate. As a result, this pathogen frequently gains competitive advantage in the rumen, due to the nutritional imbalances leading to increases in the Bacteroidetes population (Nocek, 1997; Tadepalli et al., 2009). Interestingly, the uncultivated genus “BS11 gut group” has been reported as the dominating genus in the rumen of five moose (*Alces alces*) that died in Minnesota and one moose that died in Columbus Zoo (Solden et al., 2017). It has been also demonstrated that bacteria of order Bacteroidales are frequently involved in the pathogenesis in mammals (Kraipowich et al., 2000; Hardham et al., 2008); for example, *Odoribacter denticanishas* been reported as the inducer of abdominal aposteme in human (Hardham et al., 2008). Furthermore, *Bacteroides fragilishas* been isolated from aborted bovine fetuses with symptoms of placentitis and bronchopneumonia (Kraipowich et al., 2000).

It appears safe to suggest that the seasonal shifts in ruminal concentrations of certain micro-organisms in our study were primarily conditioned by changes in the content of crude fiber in the diets and in the chemical composition of cell walls of feed ingredients with different seasonal availability. The winter–spring diets in our study contained significantly more crude fiber, in comparison to summer–autumn diets (Table 1). In addition, it is well-known that cell walls of Embryophyta (dominating the summer–autumn diets) contain cellulose (34–68%), hemicellulose (34–60%), and lignin (5–17%) (Van Soest, 1982); while the basic components of cell walls in lichens (dominating the winter–spring diets) are hemicellulose and lichenin (Culberson, 1969). Earlier, Solden et al. (2017) performed a metagenomic analysis of ruminal microbiota and metabolomic analysis of ruminal digesta in Alaskan moose (*Alces gigas*) in different seasons, and demonstrated that the concentrations of four OTUs within the microbiome were the most affected by the winter diet, compared to the spring diet, and that the concentration of the “BS11 gut group” in winter was 800-fold higher. The results of this study evidenced that the species from “BS11 gut group” degrade the monomers of hemicellulose (*i.e*., xyloses, fucoses, mannoses, and rhamnoses) producing acetate and butyrate. The authors concluded that ruminal concentration of “BS11 gut group” can increase in response to diets with higher hemicellulose content.

The seasonal changes in diets likely shift the level of consumption of energy and nutrients by the host reindeer, as well as by the microbiota of their gastrointestinal tract (Bergmann et al., 2015; Trosvik et al., 2018). Microbial fermentation is most likely a factor participating in the compensation for the detrimental effects of nutritively scarce winter–spring diets, decreased energy consumption due to the restriction in feed resources, and the consequences of exposure to extremely low ambient temperatures in reindeer. It has been recently demonstrated that the intestinal microbiota in mammals promotes the adaptation of hosts to low temperatures and regulates energy metabolism (Chevalier et al., 2015). *Vice versa*, the increased susceptibility to diseases and decreased livability under conditions of limited feed resources in the winter–spring season can result from an absence of reaction by the intestinal microbiome to these seasonal changes (Gogarten et al., 2012).

Despite the stability of ruminal communities in reindeer at different ages in our study, certain minor phyla and genera underwent age-related alterations. The differences in these ruminal communities between calves and adults can pre-determine the different levels of their adaptation to the severe conditions of the habitat. Previous studies on ruminal microbiomes have also demonstrated certain age-related alterations: Jami et al. (2013) found such alterations in cattle. In their study, the concentrations of strictly and optionally aerobic taxons were decreased, while the concentrations of anaerobic taxa increased with age. Our previous studies have also evidenced age-related changes in the ruminal microbiota of reindeer from the YNAD during the summer–autumn season (Laishev et al., 2020).

### Predicted metabolic pathways of ruminal microbiota

The PICRUSt2 data and analysis with the use of KEGG demonstrated that the potentially functional metabolic pathways of the ruminal microbiota in reindeer were dominated by pathways related to the metabolism of energy, protein, lipids, the synthesis of VFA (including lactate), degradation and catabolism of carbohydrates, synthesis of nucleic acids, nucleotides, nucleosides, cofactors, enzymes, vitamins, and cell wall elements. These data agree with the data of the taxonomic analysis of ruminal microbiota, being dominated by species from phylum Firmicutes and phylum Bacteroidetes, which degrade complex saccharides to VFA, fats, and proteins (Jami et al., 2013). These results affirm the statement that the gastrointestinal microbiota can regulate a wide range of metabolic processes in the host, including energetic homeostasis, metabolism of glucose, lipids, and so on (Sonnenburg & Bäckhed, 2016).

Our data also reaffirmed the conclusion that the ruminal microbiota in different species (including reindeer) can perform certain metabolic functions, including those which cannot be performed by the host, such as the degradation of complex polysaccharides (Macfarlane & Macfarlane, 2012). The metabolism of the microbiota can result in the production of microbial metabolites, serving as the intermediate substrates and/or signaling molecules for the host; for example, VFAs produced in the rumen can serve as the substrates for the production of energy, lipogenesis, and gluconeogenesis (Bergman, 1990; den Besten et al., 2013). On the other hand, VFAs can act as signaling molecules which are affine to certain receptors of the host, such as GPR43/FFAR2 and GPR41/FFAR3 (Tolhurst et al., 2012; Kimura et al., 2013), thus significantly affecting metabolism and its homeostasis, as well as the health status of the host. These findings are important for the clarification of bacterially mediated physiological mechanisms affecting the host metabolism in ruminants.

Earlier, Pope et al. (2012) studied the diversity of enzymatic mechanisms of ruminal transformation of lignocellulose biomass from the winter diets in adult female Svalbard reindeer (*Rangifer tarandus platyrhynchus*). Their reconstruction of the metabolic pathways of the ruminal microbiota revealed multiple polysaccharide utilization loci and over 20 families of glycosidehydrolases and other enzymes targeted at different carbohydrates, including cellulose, xylan, and pectin.

### Predicted metabolic pathways of ruminal microbiota: the effects of season and age

It is interesting to note that a large part of the pathways that differed between the samples were related to the biosynthesis of different homologues of vitamin K. This is important, as vitamin K has been reported to significantly affect certain biological functions in macroorganisms including the regulation of transcriptional activity by nucleic receptors. The activity of different homologues can vary, depending on different factors, including the chemical structure of the alkyl side chain (Hirota & Suhara, 2019). Vitamin K and its homologues are known to improve the functions of the cardiovascular system, kidney, and brain (Nakagawa et al., 2010); as well as activating metabolism of the bone tissues (Booth, 2012). Additionally, certain authors have suggested the antioxidative properties of vitamin K (Mukai et al., 1993); for example, paralogic enzyme VKORC1L1, which is expressed in various different tissues, reportedly mediates a vitamin K-dependent intracellular antioxidative function in cell membranes (Westhofen et al., 2011). The most studied function of vitamin K is its role as a cofactor in the activation of vitamin K-dependent blood clotting factors (Danziger, 2008). Vitamin K has also been reported to affect the synthesis of a protein inhibiting the mineralization of the extracellular matrix and participating in vascular remodeling (Nelsestuen & Suttie, 2002; Schurgers et al., 2005). It has been established that deficiencies of menaquinone or phylloquinone in animals can result in hemorrhages (Dam, 1935; Dam, Schønheyder & Tage-Hansen, 1936). As such, the rumen microbiota is a key factor in shaping the biochemical profile of the diet and, therefore, its impact on host health and disease.

These pathways tended to be more active in the summer–autumn season, compared to winter–spring, both in calves and adults; and in adults compared to calves in the summer–autumn season. There is likely a deficit of vitamin K in the winter–spring season in all ages of reindeer, where calves are probably more deficient than adults.

The main dietary source of vitamin K in plant diets for ruminants is vitamin K1, from the chlorophyll of Embryophyta (Booth, 2012), while menaquinones are primarily synthesized by symbiotic bacteria (Bentley & Meganathan, 1982). We analyzed the profiles of certain predicted metabolic pathways for the synthesis of vitamin K homologues by the ruminal microbiota using the MetaCyc database and reaffirmed the statement that the main precursor for different vitamin K analogues is chorismic acid (Hagervall et al., 1990). This acid is known to be synthesized by bacterial cells, through the shikimate pathway (Barker & Frost, 2001; Dosselaere & Vanderleyden, 2001). It has been also found in the chloroplasts of Embryophyta (Santos Sánchez et al., 2019). Lichens have been reported to contain no chorismic acid (Storeheier et al., 2002; Kosanic et al., 2014), likely due to the fact that the expression of phenolic compounds is strongly promoted by ultraviolet radiation (Santos Sánchez et al., 2019).

Therefore, it would be safe to suggest that the activation of the metabolic pathways relating to the synthesis of vitamin K by the ruminal microbiota in reindeer in the summer–autumn season, compared to the winter–spring season, found in our study was related to the dietary composition; namely, due to the summer-related increase in the dietary content of vitamin K precursors.

The significant seasonal increase in the activity of the pathways related to the biosynthesis of a chlorophyll precursor (chlorophyllide) in the summer–autumn season in calves and adults observed in our study can be related to the concomitant increase in the ruminal concentration of species from phylum Cyanobacteria. These oxygenic phototrophic bacteria have been reported to synthesize enzymes catalyzing the reduction in protochlorophyllide to chlorophyllide, including light-dependent NADPH: Protochlorophyllide oxidoreductaseand dark-operative protochlorophyllide oxidoreductase (Kopečná, Sobotka & Komenda, 2013). It means that the rumen microbial community composition and metabolic pathways change across seasons due to variation in temporal availability of food resources.

It is interesting to note that the overall metabolic potential of the ruminal microbiota in reindeer was more active in the summer–autumn season, compared to winter–spring; as well as in adults, compared to calves, in the summer–autumn season. Season-and age-related changes were found in the metabolic pathways of the biosynthesis of VFA, metabolism of protein, lipids, and energy, pathogenesis, carbohydrate degradation, methanogenesis, transformation of butanedioltopyruvate, degradation of neurotransmitters, and lactic acid fermentation.

These changes in the predicted metabolic pathways were correlated with the changes in the concentrations of certain genera from Bacteroidetes and Actinobacteriа, phylum Firmicutes, etc. in the same groups; for example, activation of the pathways of VFA biosynthesis could be related to the concomitant increase in the ruminal concentrations of certain Firmicutes genera (*Moryella*, *Shuttleworthia*, uncultivated genus from family Lachnospiraceae) producing large amounts of these metabolites (Downes et al., 2002; Cepeljnik et al., 2003; Tarakanov, 2006; Carlier, K’ouas & Han, 2007). The activation of energy metabolism, including the glycolysis pathway P341-PWY, could be related to the increase in the concentrations of cellulolytic bacteria, including family Oscillospiraceae etc. Glycolysis is a universal pathway of the catabolism of glucose, one of three possible pathways of glucose oxidation (together with pentose–phosphate and Entner–Doudoroff pathways) in living cells (Harden & Young, 1908). The Oscillospiraceae species and certain other ruminal cellulolytic bacteria reportedly produce a wide range of glycosidehydrolases with multiple different functions, including the degradation of cellulose and hemicellulose. Their activity serves as a basis for the biological system of degradation of glycoside bonds with the release of glucose molecules (Pascual et al., 2020).

The seasonal activation of predicted pathways related to the metabolism of lipids could be associated with the ability of certain ruminal bacteria for lipolysis and biohydration of unsaturated to saturated fatty acids (Harfoot & Hazlewood, 1997). Certain Selenomonadaceae species (whose ruminal concentration in reindeer was found to increase in the summer–autumn season in our study), including *Anaerovibrio lipolytica*, are the lypolytics synthesizing esterase and lipase (Harfoot, 1978). The function of lipase is the entire hydrolysis of triglycerides to free fatty acids and glycerol, as well as small amounts of their intermediates, di-and mono-acylglycerols (Hawke & Silcock, 1970). The released glycerol, in turn, undergoes prompt enzymatic transformation to propionic acid (Garton, Lough & Vioque, 1961). Christensenellaceae species (again, more abundant in the summer–autumn season in adult reindeer) can also affect certain aspects of lipid metabolism (Everard et al., 2013).

The seasonal shifts in the composition of ruminal microbiota and in the activities of its predicted metabolic pathways were evidently related to the seasonal changes in dietary composition; for example, the activation of the pathways related to the metabolism of lipids and energy in the summer–autumn season could be associated with the increase in dietary crude fat content, activation of the pathways of protein metabolism with increase in crude protein content, and so on.

The increased predicted metabolic activity of ruminal microbiota in adult reindeer, in comparison to calves (regardless of season), could also be related to the changes in its composition.

Earlier, Henderson et al. (2015) analyzed 742 samples of ruminal content of 32 ruminant species from 35 countries of the World and concluded that season-and age-related changes in the composition of ruminal microbiota can significantly contribute to the concomitant changes in the metabolism of the microbiota and the host. The study by Salgado-Flores et al. (2016) on reindeer fed with lichens *vs*. standard pelleted compound feed was primarily oriented to the investigation of ruminal methanogenesis; the pathways related to the metabolism of pyruvate, fatty acids, starch, saccharose, and polysaccharides were among the most diet-affected predicted metabolic pathways of the ruminal microbiota in their study.

The alterations in the metabolic activity of the gastrointestinal microbiota (including ruminal) in reindeer can be regarded as a part of the system of mechanisms for the compensation of decreased energy and nutrient consumption in winter (Springer et al., 2017).

## Conclusions

The nutritional value of winter–spring and summer–autumn diets of reindeer were found to significantly differ: Crude fiber content in the winter–spring diet was higher by 22.4%, compared to summer–autumn, indicating possibly poorer digestibility of the winter–spring diet and a necessity for the adaptation of the ruminal microbiota to this seasonal change. A total of 580,878 sequences of microbial 16S rRNA in the rumen were identified: 22 bacterial phyla were found, with phyla Bacteroidetes and Firmicutes being the dominating taxa (up to 48.1% ± 4.30% and 46.1% ± 4.80%, respectively), while only two archaeal phyla presented as minor communities (no more than 0.54% ± 0.14% totally). Estimation of the network of microbial associations (by SPIEC-EASI) revealed the bacterial species with the highest number of associations with other species (concentrators), belonging to classes Bacteroidia (Bacteroidetes) and Negativicutes (Firmicutes). Our results confirmed the important role of the ruminal microbiota in digestion and metabolism. The analysis of biodiversity of the ruminal microbiota in calves *vs*. adult animals consuming winter–spring or summer–autumn diets revealed no significant differences in the Shannon’s and Simpson’s indices. The percentages of the dominating taxa (Bacteroidetes and Firmicutes) were also not affected by age or season, indicating evolutionally advanced adaptation of this stable community to seasonal changes in diet. However, despite the stability of this dominant community, significant changes in certain minor communities at the phyla and genera levels were found, with seasonal changes being more significant than age-related. The percentage of phylum Actinobacterta significantly increased (19.3-fold) in the summer–autumn season, compared to winter–spring in adults, while the percentage of phylum Cyanobacteria differed by up to seven-fold in adults and calves. The seasonal changes in the composition of ruminal microbiota can improve the ability of reindeer to withstand seasons with poor feed quality and low nutrient availability. The results of PICRUSt2 simulation and its analysis with the use of KEGG revealed 257 predicted metabolic pathways in the ruminal microbiota: 41 pathways were significantly influenced by season and/or age, including the processes of synthesis of vitamins, volatile fatty acids, and pigments; metabolism of protein, lipids, and energy; pathogenesis, methanogenesis, butanediol to pyruvate biosynthesis, cell wall biosynthesis, degradation of neurotransmitters, lactic acid fermentation, and biosynthesis of nucleic acids. A large part of these changeable pathways (13 of 41) was related to the synthesis of different vitamin K homologues.

The results obtained serve to improve our knowledge on the structure and possible metabolic pathways of the ruminal microbiota in reindeer—in particular, related to the provision of the host with energy and nutrients—in relation to seasonal changes. Further research is required to clarify the mechanisms underlying the complex inter-relationships between the gastrointestinal microbiota and the host. Future studies may include RNA sequencing of the ruminal microbiome, as investigation of the transcriptional activity of the microbiome can contribute to the quantitative profiling of the metabolic potential of ruminal microbiota and detail its functional activity, as predicted in our study at the microbiome level.

## Supplemental Information

10.7717/peerj.12389/supp-1 Supplemental Information 1 ARRIVE 2.0 checklist.

10.7717/peerj.12389/supp-2 Supplemental Information 2 Biochemical composition of pasture diets of reindeer. The raw data is showing the raw measurements of biochemical composition of summer–autumn (CS and AS treatments) and winter–spring (CW and AW treatments) pasture diets of calves and adult reindeer.

## Additional Information and Declarations

### DNA Deposition

The following information was supplied regarding the deposition of DNA sequences: The 16S rRNA gene sequences are available at the National Center for Biotechnology Information (NCBI) Sequence Read Archive (SRA): PRJNA576999.

### Data Availability

The following information was supplied regarding data availability: The raw measurements of the biochemical composition of summer–autumn (CS and AS treatments) and winter–spring (CW and AW treatments) pasture diets of calves and adult reindeer are available in the Supplemental File.
